# The Spread Distance of Local Anesthetics in the Rectus Sheath Block in Children Aged 0-8 Years: A Retrospective Study

**DOI:** 10.7759/cureus.52084

**Published:** 2024-01-11

**Authors:** Hazar Kokten, Filiz Uzumcugil

**Affiliations:** 1 Anesthesiology and Reanimation, Hacettepe University School of Medicine, Ankara, TUR

**Keywords:** local anesthetic, abdominal hernia, laparoscopy, regional anesthesia, injection, children

## Abstract

Background

The spread distance of local anesthetic (LA) in the rectus sheath block (RSB) should cover the planned surgical incision. However, there is limited data regarding the spread distance that can be covered by a certain volume in children. In this study, we aimed to investigate the spread distance of a particular volume of LA from a single injection point.

Methodology

This study included children aged 0-8 years (n = 41) who underwent umbilical or epigastric hernia repair, laparoscopic surgery, and surgeries via small midline incisions. The spread distances, which were measured via ultrasound guidance immediately after the block to ensure coverage of the entire planned surgical incision, were obtained from prospectively collected data about RSB. The spread distances in the craniocaudal direction on the right and left sides were compared and assessed for correlations with age, weight, LA volume, and sex. The need for a supplemental dose of LA in case of an incision exceeding the spread distance was also obtained from the records.

Results

The spread distances in the cranial and caudal directions from the injection point were 3.28 ± 1.04 cm and 3.74 ± 1.35 cm on the right (p = 0.066) and 3.44 ± 1.02 cm and 3.44 ± 1.33 cm on the left (p > 0.999), respectively. The total spread distances in the craniocaudal direction on the right and left were 5.55 ± 0.95 cm and 5.72 ± 1.28 cm in patients aged 0-2 years and 7.25 ± 1.92 cm and 7.39 ± 1.81 cm in patients aged at 2-8 years, respectively. The increase in the spread distance correlated with age, weight, and LA volume. None of the patients required a supplemental dose of LA, as the single-point injections covered the planned surgical incisions in all patients.

Conclusions

Similar spread distances in the cranial and caudal directions from a single injection point can be obtained with 0.5 mL/kg of the LA in 0-8-year-old children. The total spread distance in the craniocaudal direction was approximately 5 cm and 7 cm in children aged 0-2 years and 2-8 years, respectively.

## Introduction

The rectus sheath block (RSB) can be used to provide analgesia for anterior abdominal wall surgeries such as umbilical and epigastric hernia repair, laparoscopic surgery, and midline laparotomy procedures. The spread of the local anesthetic (LA) solution in the rectus sheath should cover the entire surgical incision to achieve the optimal effect of the RSB [1]. The factors affecting this spread have been addressed in previous studies including both anatomical and clinical studies [1-6]. The anatomical studies included animal cadavers and adult human cadavers, whereas the clinical studies mostly included adult patients [1,2-4,7,8]. The limited clinical studies on the effect of RSB in children included pediatric patients of a large age range, leading to a heterogeneous group in terms of anthropometric measures [1,5,9,10-12]. The optimal LA volume to be used as well as its concentration and dose remain controversial; however, some studies have suggested a volume of 10-30 mL in adults and 0.1-0.5 mL/kg in children [13-15]. Previously, determining the LA volume according to the anthropometric measures of the patients was considered suitable because the body mass index is a predictor of the spread pattern of the LA solution in RSB in adult patients [8]. In addition to determining the LA volume, the injection point and number of injections required were studied. Knowing the spread distance that can be achieved with a single injection could guide in deciding whether more than one injection is required to cover the entire surgical incision [2]. Our experience on RSB grew with the evaluation of the spread distance of LA within a total length of the abdominal wall in mid-line from xiphoid to pubis. In our observations, with 0.5 mL/kg of LA solution injected from a single injection point, two dermatomes can be covered, not more. However, as identifying dermatomal levels may be unreliable within a study population of rapidly growing age and the quality of the RSB mainly depends on the coverage of surgical incision, our primary goal was to evaluate the actual spread distance of the LA solution in the rectus sheath with a single injection. Hence, our study depends on the principle that recommends the coverage of the entire surgical incision with the injected LA for the RSB to be effective. Our secondary goal was to investigate the relationship between the spread distance and age, sex, weight, and LA volume in the pediatric population.

This work has been sent as an abstract to the European Society of Anaesthesiology and Intensive Care (ESAIC) 2024.

## Materials and methods

After Institutional Ethical Board approval was obtained (approval number: GO 22/1135-2022/22-26), patients aged 0-8 years who received RSB for elective surgical procedures with a midline incision between April 1, 2022, and October 1, 2022, were included in the study. The anesthetic records of these patients were reviewed to obtain data on demographics, surgical procedures, and the dose, concentration, and volume of the LA solution. The spread distances were obtained from a prospectively collected dataset which was recorded to ensure that the spread distance of the LA solution correlated with the planned surgical incision. The lack of data was a criterion for exclusion.

According to the routine practice at our hospital, all patients had undergone a standardized anesthetic approach. All patients were premedicated by administering intranasal (0.2-0.3 mg/kg), oral (0.5-0.75 mg/kg), or intravenous (0.02-0.03 mg/kg) midazolam before admission to the operating room. The patients with an intravenous line received anesthetic induction with propofol, whereas all others received inhalational induction using sevoflurane. All patients received fentanyl for induction and sevoflurane supported by remifentanil infusion for maintenance. Rocuronium was used for neuromuscular blockade if required.

**Figure 1:**
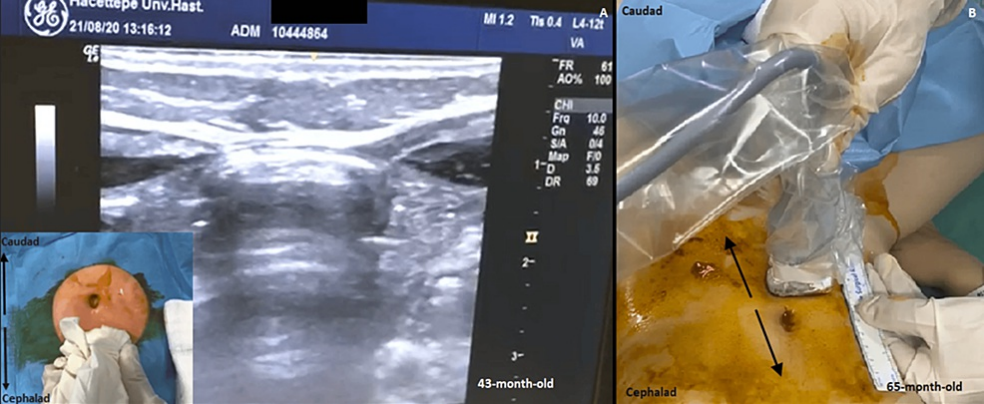
The measurement of spread distance by sliding the ultrasound probe toward the cephalad and caudad directions from the injection point. (A) Epigastric hernia repair of a patient at 43 months of age with the ultrasound probe at the injection point. (B) Laparoscopic surgery of a patient at 65 months of age showing the measurement technique.

According to our protocol, if the planned surgical incision did not exceed approximately the two dermatome level, the RSB was administered with a single injection from a needle entry point at the midpoint of the planned surgical incision using the in-plane lateral-to-medial technique under ultrasound guidance. First, the length of the planned surgical incision was marked by the surgeons for us to determine the midpoint as our single injection point. Then, the RSB was administered in all patients using 0.5 mL/kg/side bupivacaine at a concentration of 0.25%. After injection, the spread distance was measured to improve the quality of the block. The spread of the LA between the rectus muscle and posterior sheath was assessed by using ultrasound. The spread distance in the caudal and cranial directions from the needle entry point was measured using a ruler to check whether the spread covered the planned surgical incision (Figure 1). In case of an inadequate spread, an additional LA dose was injected to cover the incision. This supplementary LA injection was 0.25 mL/kg at a dose of 0.5 mg/kg of bupivacaine to avoid exceeding the recommended maximum dose of 3 mg/kg.

The total spread distances in the craniocaudal direction were compared between the left and right sides. Additionally, the cranial and caudal spread distances from the injection point were compared between the two sides. The correlations between the total spread distances in the craniocaudal direction and age, weight, and LA volume were assessed. To evaluate the influence of the anthropometric features on the LA spread, patients were grouped into age groups of 0-2 years (0<x≤2 years) and 2-8 years (2<x≤8 years) assuming that the patients in each group had more similar width of dermatomes. Sex differences in the groups were also assessed.

Data analysis was performed using SPSS version 25 (IBM Corp., Armonk, NY, USA). The Shapiro-Wilk test was used to determine whether the continuous numerical variables showed a near-normal distribution. Descriptive statistics are presented as mean ± standard deviations for continuous numerical variables and as numbers and percentages for categorical variables. A dependent t-test was used to determine whether there was a statistically significant change in the average amount of spread distances by direction (cranial vs. caudal) and sides (right vs. left). The significance of the correlation between the continuous numerical variables was investigated using Pearson’s moment-product correlation coefficients. Student’s t-test was used to determine whether there was a statistically significant difference in the mean amount of spread distance by gender. Unless otherwise stated, p-values <0.05 were considered statistically significant. However, Bonferroni correction was used to control for type I errors in all possible multiple comparisons.

## Results

We reviewed the anesthetic records of children aged 0-8 years, who underwent elective surgery involving a midline incision on the abdominal wall between April 1, 2022, and November 1, 2022. This study included 41 patients. Table 1 shows the demographics and types of surgical procedures performed.

**Table 1:** The demographic data and the types of surgical procedures.

	N = 41
Age (year) (mean ± SD)	4.4 ± 2.3
Gender	
Male	19 (46.3%)
Female	22 (53.7%)
Weight (kg)	18.5 ± 7.5
Types of surgical procedures	
Laparoscopy	16 (39.0%)
Umbilical hernia	11 (26.8%)
Epigastric hernia	6 (14.6%)
Others	10 (24.4%)

Spread distances covered the planned surgical incision in all of our patients, none of whom required a supplemental LA injection.

The mean volume of the LA solution administered was 8.8 ± 3.4 mL. The spread distances from the injection point on the left and right sides in the cranial and caudal directions are shown in Table 2. The difference between the spread distances in the cranial and caudal direction was 0.00 ± 1.44 cm and 0.46 ± 1.57 cm on the left and right sides, respectively (p = 0.015) (Table 2). However, the actual spread distances in the cranial and caudal directions on the left and right sides were similar. The total spread distances in the craniocaudal direction on the left and right sides were 6.88 ± 1.88 cm and 7.02 ± 1.83 cm, respectively (p = 0.501) (Table 2). For the sub-groups of ages, the total craniocaudal spread distances on the left and the right sides were 5.55 ± 0.95 cm and 5.72 ± 1.28 cm in patients aged 0<x≤2 years and 7.25 ± 1.92 cm and 7.39 ± 1.81 cm in those aged 2<x≤8 years, respectively (Table 2).

**Table 2:** The spread distances (cm) in the cranial and caudal directions on the left and right sides. ^†^: the comparisons between cranial spread distances on the left and right sides, as well as the caudal spread distances on the left and right sides; ^‡^: the comparisons between cranial and caudal spread distances on the left and right sides; ^ɤ^: the comparisons between total spread distances on the left and right sides between age groups; ^¶^: p < 0.0125 was statistically significant according to Bonferroni correction; ^¥^: p < 0.025 was statistically significant according to Bonferroni correction; ^§^: p < 0.05 was statistically significant; ^1^: the differences between left caudal and right caudal, the differences between left cranial and right cranial; ^2^: the differences between left caudal and left cranial, the differences between right caudal and right cranial; ^3^: total craniocaudal spread distances on the left and right sides.

	Left	Right	P-value^†^	Difference in spread distances on the left and right sides^1^
Caudal	3.44 ± 1.33	3.74 ± 1.35	0.025^¶^	-0.30 ± 0.83
Cranial	3.44 ± 1.02	3.28 ± 1.04	0.299^¶^	0.16 ± 0.96
P-value^‡^	>0.999^¶^	0.066^¶^		0.015^¥^
Difference in spread distances in cranial and caudal directions^2^	0.00 ± 1.44	0.46 ± 1.57	0.015^¥^	-0.46 ± 1.57
Total spread distances^3^	6.88 ± 1.88	7.02 ± 1.83	0.501^§^	-0.14 ± 1.38
0-2 years of age	5.55 ± 0.95	5.72 ± 1.28	0.700^¥^	
2-8 years of age	7.25 ± 1.92	7.39 ± 1.81	0.583^¥^	
P-value^ɤ^	0.015^¥^	0.014^¥^		

The spread distances were correlated with age, weight, and LA volume (Table 3).

**Table 3:** . The correlation coefficients (CCs) between the craniocaudal spread distance and age, weight, and volume of the local anesthetic solution. ^†^: Pearson’s product-moment correlation coefficient test; ‡: p < 0.0125 were significant according to Bonferroni correction; ^¶^: p < 0.025 were statistically significant.

	Left		Right	
	CC	P-value^†^	CCı	P-value^†^
Caudal				
Age	0.502	<0.001^‡^	0.489	<0.001^‡^
Weight	0.493	<0.001^‡^	0.419	0.006^‡^
Volume	0.513	<0.001^‡^	0.481	<0.001^‡^
Cranial				
Age	0.438	0.004^‡^	0.449	0.003^‡^
Weight	0.505	<0.001^‡^	0.444	0.004^‡^
Volume	0.529	<0.001^‡^	0.418	0.007^‡^
Total				
Age	0.592	<0.001^¶^	0.616	<0.001^¶^
Weight	0.623	<0.001^¶^	0.562	<0.001^¶^
Volume	0.649	<0.001^¶^	0.592	<0.001^¶^

The differences between spread distances in the cranial and caudal directions were similar on both sides (p > 0.0125) (Table 4). These differences did not correlate with age, weight, or LA volume.

**Table 4:** Correlation coefficients between the differences of spread distances and age, weight, and volume of the local anesthetic solution. ^†^: Pearson’s product-moment correlation coefficient test; p < 0.0125 were significant according to Bonferroni correction.

	Correlation coefficient	P-value^†^
Age		
Caudal left – Caudal right	0.009	0.954
Cranial left – Cranial right	-0.025	0.879
Cranial left – Caudal left	-0.153	0.340
Cranial right – Caudal right	-0.120	0.455
Weight		
Caudal left – Caudal right	0.108	0.502
Cranial left – Cranial right	0.051	0.751
Cranial left – Caudal left	-0.097	0.546
Cranial right – Caudal right	-0.063	0.695
Volume		
Caudal left – Caudal right	0.040	0.803
Cranial left – Cranial right	0.105	0.513
Cranial left – Caudal left	-0.098	0.540
Cranial right – Caudal right	-0.133	0.406

The sex difference did not correlate with the spread distances (Table 5). The differences between the spread distances in the cranial and caudal directions on the left side were similar between male and female children. The cranial spread distance was 0.18 ± 1.42 cm more than the caudal spread distance on the right side in male children, whereas the caudal spread distance was 1.02 ± 1.51 cm more than the cranial spread distance in female children (Table 5).

**Table 5:** The cranial and caudal spread distances (cm) with differences in spread distances between the cranial and caudal directions on both sides in males and females. Data were presented in mean ± standard deviation (cm). ^†^: Student’s t-test; ^‡^: p < 0.0125 were significant according to Bonferroni correction; ^¶^: p < 0.025 were statistically significant.

	Male (n = 19)	Female (n = 22)	P-value^†^
Caudal			
Left	3.16 ± 1.33	3.68 ± 1.30	0.212^‡^
Right	3.34 ± 1.33	4.09 ± 1.29	0.075^‡^
Cranial			
Left	3.63 ± 0.95	3.27 ± 1.07	0.266^‡^
Right	3.53 ± 0.90	3.07 ± 1.14	0.166^‡^
Total			
Left	6.79 ± 1.86	6.95 ± 1.94	0.783^¶^
Right	6.87 ± 1.79	7.16 ± 1.90	0.619^¶^
Difference in spread distances in cranial and caudal directions			
Caudal left – Caudal right	-0.18 ± 0.93	-0.41 ± 0.75	0.397
Cranial left – Cranial right	0.10 ± 0.89	0.20 ± 1.04	0.747
Cranial left – Caudal left	0.47 ± 1.40	-0.41 ± 1.38	0.049
Cranial right – Caudal right	0.18 ± 1.42	-1.02 ± 1.51	0.012

## Discussion

In our study, we observed that similar spread distances could be obtained in the cranial and caudal directions on the left and right sides of the anterior abdominal wall by injecting 0.5 mL/kg of the LA solution from a single injection point. The total spread distance in the craniocaudal direction on the left and right sides were 5.55 ± 0.95 and 5.72 ± 1.27 cm in children aged 0<x≤2 years and 7.25 ± 1.91 and 7.42 ± 1.77 cm in those aged 2<x≤8 years, respectively. No additional dose of LA was required.

Studies addressing the effectiveness of RSB have highlighted that the LA injection should cover the entire surgical incision [1]. Hence, the spread distance in the craniocaudal direction and the factors affecting this spread have gained interest in anatomical studies [1-7]. However, the spread in the mediolateral direction has also been investigated to increase the effectiveness of the block [3,7]. The efficacy of RSB is reportedly enhanced when the LA solution is injected from the lateral to the medial direction to block the thoracoabdominal nerves before they enter the rectus muscle through their course in the lateral-to-medial and craniocaudal directions inside the sheath [3,4,8]. Anatomical studies included either adult cadavers or animals [2-4,7], with only a few studies addressing the spread patterns in actual patients [1,8]. Our study reveals the importance of addressing the spread distance of the LA solution in the rectus sheath from a single injection point with an injection from the lateral to the medial direction in children of a specific age group.

Monassero et al. investigated the spread pattern of the LA solution in RSB in adult patients [8]. They used a single fixed volume of LA for all patients and injected it from the medial to the lateral direction, which is the opposite of the recommended technique. Moreover, a categorical method was used to evaluate the spread pattern instead of a direct measure of the spread distance [8]. The authors concluded that the body mass index of the patients was the most important factor adversely affecting the spread of the LA solution; hence, determining the LA volume according to the anthropometric measurements of the patients was suggested. However, they also suggested that the effect of the RSB could be enhanced by either changing the direction of the injection or administering two injections from different points instead of a single injection [8]. In our study, the volume of LA injected was based on the weight of the patients, and the block was administered by injecting in the lateral-to-medial direction.

Similar to our study, Visoiu et al. investigated the spread distance of LA in the craniocaudal direction from a single injection point in the umbilicus in 68 children under 18 years of age [1]. The spread distance in the cranial direction was greater than that in the caudal direction on both sides of the abdominal wall. This difference could be attributed to the presence of the arcuate ligament in the caudal direction. The authors reported that the LA reached the subcostal line on the right side in 52.9% of patients and on the left side in 36.8% of patients. Hence, they emphasized that it might be more suitable to change the injection point according to the surgical incision site [1]. Our study has strengths in certain points. The age group of the children in the study by Visoui et al. [1] constituted a heterogeneous group considering the anthropometric differences among children in the 0-18-year range. In our study, the age group was 0-8 years, which could be expected to be a homogeneous group; moreover, the spread distances were also compared between subgroups of ages comprising children aged 0-2 years and 2-8 years to ensure homogeneity. Although we also used a single injection point, this point was adjusted according to the surgical incision site to target the midpoint of the planned incision. Hence, we assessed the spread distance from the injection point, not from the umbilicus, and found a similar spread distance in both the cranial and caudal directions. Therefore, we suggest that the optimum injection point for the RSB can be the midpoint of the planned surgical incision site. On the other hand, as we directly measured the spread distance of a volume of 0.5 mL/kg of LA, the data in our study may contribute to the current literature to help decide whether one or two injection points are required to cover the entire surgical incision according to its length. A similar suggestion of deciding the need for two injection points for the RSB to cover the surgical incision, based on the knowledge of the spread distance that can be obtained with a single injection, was made by Rojas et al. in their cadaver study [2].

The spread pattern of the LA was found to be correlated with the injected volume of the LA solution, as well as the age, body weight, height, and body mass index of the patient [1,8]. Injecting 0.1 mL/kg and 0.5 mL/kg from a single injection point in children has been reported when we consider studies addressing the efficacy of RSB [5,9-12]. These clinically effective volumes were also addressed in anatomical studies [1,4]. In a recent animal cadaver study, 0.25 mL/kg and 0.5 mL/kg volumes were compared for their spread patterns in the RSB, and a longer spread with higher involvement of the targeted nerves was observed with the latter than with the former [4]. We used 0.5 mL/kg as the standard dose in the procedure in our study and found that it was correlated with the LA volume, as well as the age and weight of the patient.

The difference between the cranial and caudal spread distances on the right was significantly greater than that on the left side. Although it may not have clinical significance that none of our patients required a supplemental dose, it may be worth investigating because this difference was also significant when it was evaluated concerning sex differences. The difference between the caudal and cranial spread distances was significantly greater on the right side than on the left side in female patients. Hence, investigation of the spread pattern on the right side in further studies is warranted.

Limitations

Our study is based on the principle that recommends the coverage of the entire surgical incision with the injected LA for the RSB to be effective. In our daily practice, we observed that incisions longer than approximately two dermatomes, considering the six dermatomes innervating the entire anterior abdominal wall, required more than one LA injection to cover an entire midline incision. Additionally, supplemental blocks were indicated because of the drainage catheters on the anterolateral sides of the abdominal wall. As we use more than one single injection for incisions exceeding approximately two dermatomes and supplementary blocks for surgeries including the anterolateral sites of the abdominal wall, we did not include these patients in the study. Unfortunately, we only aimed to cover the planned surgical incision and because we did not perform any measurement regarding LA injection after the surgery, we did not measure the actual length of the incisions. The coverage might have been analyzed and interpreted better if we measured and investigated their correlation, which can be a major limitation of our study. Notably, our study results could help determine whether one or two injections are required to cover a midline incision by using 0.5 mL/kg of LA solution. However, the spread distances of different volumes from a single injection point should be compared in prospective studies in children.

Another limitation of our study can be the evaluation of dermatomes. Considering the rapidly and gradually growing nature of pediatric group patients, it may not be reliable to include different age groups. However, it might be better to obtain the length of the entire midline of the abdominal wall and interpret the spread distance in terms of a ratio or percentage according to this length in more granular groups of similar anthropometric measures.

In our study, the measurements were obtained immediately after the LA injection to determine whether it covered the entire length of the planned surgical incision. However, the changes in the spread pattern over time were not addressed.

There can also be observer bias as the distance was measured using ultrasound, which needs to be mentioned as a possible limitation of our study.

## Conclusions

We observed that 0.5 mL/kg of LA from a single injection point spread to a similar distance in both cranial and caudal directions within the rectus sheath on both sides of the abdominal wall. The total spread distance was 5 cm and 7 cm in the craniocaudal direction in patients aged 0-2 years and 2-8 years, respectively. These spread distances obtained with 0.5 mL/kg of LA could guide in deciding whether one or more injections are required according to the planned surgical incision.

## References

[1] Visoiu M, Hauber J, Scholz S (2019). Single injection ultrasound-guided rectus sheath blocks for children: distribution of injected anesthetic. Paediatr Anaesth.

[2] Rojas A, McMillan DT, Allan JD (2023). Evaluating patterns of injectate spread after rectus sheath block: a cadaveric dissection study. Cureus.

[3] Seidel R, Wree A, Schulze M (2017). Does the approach influence the success rate for ultrasound-guided rectus sheath blocks? An anatomical case series. Local Reg Anesth.

[4] St James M, Ferreira TH, Schroeder CA, Hershberger-Braker KL, Schroeder KM (2020). Ultrasound-guided rectus sheath block: an anatomic study in dog cadavers. Vet Anaesth Analg.

[5] Albokrinov AA, Perova-Sharonova VM, Fesenko UA (2019). A new neurostimulator guided technique of rectus sheath block: study of feasibility and local anesthetic spread in children. Anaesthesiol Intensive Ther.

[6] Yaster M, Maxwell LG (1989). Pediatric regional anesthesia. Anesthesiology.

[7] Sakai-Tamura A, Murata H, Ogami-Takamura K, Saiki K, Manabe Y, Tsurumoto T, Hara T (2020). Course of the thoracic nerves around the umbilicus within the posterior layer of the rectus sheath: a cadaver study. J Anesth.

[8] Manassero A, Bossolasco M, Meineri M, Ugues S, Liarou C, Bertolaccini L (2015). Spread patterns and effectiveness for surgery after ultrasound-guided rectus sheath block in adult day-case patients scheduled for umbilical hernia repair. J Anaesthesiol Clin Pharmacol.

[9] Willschke H, Kettner S (2012). Pediatric regional anesthesia: abdominal wall blocks. Paediatr Anaesth.

[10] Ferguson S, Thomas V, Lewis I (1996). The rectus sheath block in paediatric anaesthesia: new indications for an old technique?. Paediatr Anaesth.

[11] Alsaeed AH, Thallaj A, Khalil N, Almutaq N, Aljazaeri A (2013). Ultrasound-guided rectus sheath block in children with umbilical hernia: case series. Saudi J Anaesth.

[12] Breschan C, Jost R, Stettner H, Feigl G, Semmelrock S, Graf G, Likar R (2013). Ultrasound-guided rectus sheath block for pyloromyotomy in infants: a retrospective analysis of a case series. Paediatr Anaesth.

[13] Courreges P, Poddevin F, Lecoutre D (1997). Para-umbilical block: a new concept for regional anaesthesia in children. Paediatr Anaesth.

[14] de Jose Maria B, Götzens V, Mabrok M (2007). Ultrasound-guided umbilical nerve block in children: a brief description of a new approach. Paediatr Anaesth.

[15] Flack SH, Martin LD, Walker BJ, Bosenberg AT, Helmers LD, Goldin AB, Haberkern CM (2014). Ultrasound-guided rectus sheath block or wound infiltration in children: a randomized blinded study of analgesia and bupivacaine absorption. Paediatr Anaesth.

